# Novel Hydrogel Membranes Based on the Bacterial Polysaccharide FucoPol: Design, Characterization and Biological Properties

**DOI:** 10.3390/ph16070991

**Published:** 2023-07-11

**Authors:** Diana Araújo, Matilde Martins, Patrícia Concórdio-Reis, Catarina Roma-Rodrigues, Maria Morais, Vítor D. Alves, Alexandra R. Fernandes, Filomena Freitas

**Affiliations:** 1 Associate Laboratory i4HB—Institute for Health and Bioeconomy, School of Science and Technology, NOVA University Lisbon, 2829-516 Caparica, Portugal; df.araujo@campus.fct.unl.pt (D.A.); mt.martins@campus.fct.unl.pt (M.M.); pc.reis@campus.fct.unl.pt (P.C.-R.); catromar@fct.unl.pt (C.R.-R.); 2 UCIBIO—Applied Molecular Biosciences Unit, Department of Chemistry, School of Science and Technology, NOVA University Lisbon, 2829-516 Caparica, Portugal; 3 UCIBIO—Applied Molecular Biosciences Unit, Department of Life Sciences, School of Science and Technology, NOVA University Lisbon, 2829-516 Caparica, Portugal; 4 i3N/CENIMAT, Department of Materials Science, Faculty of Sciences and Technology, NOVA University of Lisbon and CEMOP/UNINOVA, 2829-516 Caparica, Portugal; md.morais@campus.fct.unl.pt; 5 LEAF—Linking Landscape, Environment, Agriculture and Food Research Center, Associated Laboratory TERRA, Instituto Superior de Agronomia, Universidade de Lisboa, 1349-017 Lisboa, Portugal; vitoralves@isa.ulisboa.pt

**Keywords:** hydrogel membranes, FucoPol, ionotropic gelation, biocompatibility, anti-inflammatory activity

## Abstract

FucoPol, a fucose-rich polyanionic polysaccharide, was used for the first time for the preparation of hydrogel membranes (HMs) using Fe^3+^ as a crosslinking agent. This study evaluated the impact of Fe^3+^ and FucoPol concentrations on the HMs’ strength. The results show that, above 1.5 g/L, Fe^3+^ concentration had a limited influence on the HMs’ strength, and varying the FucoPol concentration had a more significant effect. Three different FucoPol concentrations (1.0, 1.75 and 2.5 wt.%) were combined with Fe^3+^ (1.5 g/L), resulting in HMs with a water content above 97 wt.% and an Fe^3+^ content up to 0.16 wt.%. HMs with lower FucoPol content exhibited a denser porous microstructure as the polymer concentration increased. Moreover, the low polymer content HM presented the highest swelling ratio (22.3 ± 1.8 g/g) and a lower hardness value (32.4 ± 5.8 kPa). However, improved mechanical properties (221.9 ± 10.2 kPa) along with a decrease in the swelling ratio (11.9 ± 1.6 g/g) were obtained for HMs with a higher polymer content. Furthermore, all HMs were non-cytotoxic and revealed anti-inflammatory activity. The incorporation of FucoPol as a structuring agent and bioactive ingredient in the development of HMs opens up new possibilities for its use in tissue engineering, drug delivery and wound care management.

## 1. Introduction

Hydrogel membranes (HMs) are very interesting structures that combine thin membranes’ permeability and porous architecture with the water absorption ability and dynamic mechanical properties of hydrogels. This combination allows a controlled diffusion of the molecules because permeation is limited through the bulk hydrogel, and the permeation rate depends on the size and hydrophobicity properties of the molecule. This combination also allows an enhancement in gas permeation by the water uptake ability [1]. Thus, these unique features make HMs promising materials for various applications, such as antifouling/antimicrobial coatings [2], transdermal delivery systems [3] and wound dressings [4]. Among several methods used for their fabrication, including sintering [5], electrospinning [6] and molecular imprinting [7], casting is a widely used technique due to its simplicity and low cost [1]. In general, this method involves polymer dissolution followed by a crosslinking step [8,9] and pouring on the casting plate. Crosslinking can be physically or chemically established via non-covalent or covalent interactions. Physical crosslinking might involve hydrogen bonds, van der Waals forces, chain entanglements and hydrophobic or ionic interactions, which lead to the formation of fragile and reversible hydrogels due to the weakness of these interactions [10].

Ionic interactions, present in the so-called ionotropic gelation, occur through the crosslinking of polymeric chains with ions, usually the cation-mediated gelation of negatively charged polysaccharides [11,12]. Ionotropic hydrogel formation is induced by divalent (e.g., Ca^2+^, Pb^2+^, Cu^2+^) or trivalent (e.g., Fe^3+^, Al^3+^) cations under different types of binding mechanisms. For example, divalent cations typically adopt the so-called egg-box model characterized by two antiparallel polyuronate chains forming egg-box dimers with Ca^2+^ that form multimers by aggregating laterally [13]. Although the binding mechanism of trivalent cations, namely Fe^3+^, remains under investigation, two gelation models have been proposed: (1) the coordination of the metal cation by the polysaccharide, resulting in spatially separated Fe^3+^ centers along with polysaccharides, or (2) the colloidal model, which suggests the production of oxyhydroxide colloids that are stabilized by polysaccharide chains, preventing their aggregation and keeping the colloids in the hydrogel matrix [14]. Although these models have been proposed for crosslinking by metal coordination in alginate hydrogels, several other polysaccharides, such as xanthan gum [15], konjac glucomannan [16], carboxymethyl cellulose [17] and succinoglycan [18], have been used to fabricate ionotropic hydrogels using Fe^3+^ as a crosslinker. The use of polysaccharides in hydrogel fabrication presents several advantages due to their biocompatibility, biodegradability and non-toxicity, which make them attractive biomaterials to be used in the biomedical field [19,20], particularly in topical applications [21,22].

FucoPol is a fucose containing polysaccharide, characterized by a high molecular weight (1.7–5.8 × 10^6^ Da), that is synthesized by *Enterobacter* A47 (DSM 23139). It is composed of fucose (32–36 mol%), glucose (28–37 mol%) and galactose (25–26 mol%) as neutral sugars, glucuronic acid (9–10 mol%) as an acidic sugar and acetate (3.5–6.8 wt.%), pyruvate (3.7–14 wt.%) and succinate (0.6–3 wt.%) as acyl groups [23]. Due to its intrinsic biological activity that includes antioxidant [24] and wound healing abilities [25], FucoPol is a promising biopolymer to be exploited for biomedical applications. Moreover, FucoPol possesses an anionic character given by the presence of glucuronic acid, succinyl and pyruvyl, which opens up the possibility to interact with ions and other charged macromolecules [23]. Recently, the interaction between FucoPol and several multivalent cations was studied, and the cation-mediated gelation ability of FucoPol was demonstrated [26]. In that procedure, trivalent Fe^3+^ cations were successfully used to crosslink polymeric functional groups, forming stable and non-cytotoxic hydrogel beads.

In this study, FucoPol was used for the first time to prepare HMs using Fe^3+^ as a crosslinker. The impact of FucoPol and Fe^3+^ concentration on the strength of the structures was evaluated, and the optimal conditions were selected to prepare the HMs. Furthermore, the HMs thus obtained were physically and chemically characterized in terms of their morphology and mechanical and swelling properties, and they were evaluated for their cytotoxicity and anti-inflammatory activity.

## 2. Results and Discussion

### 2.1. Fabrication of FucoPol HMs

#### 2.1.1. Effect of Fe^3+^ Concentration

The polyanionic character of FucoPol, given by the macromolecule’s negatively charged groups, namely glucuronic acid and the acyl groups pyruvyl and succinyl, allowed the formation of physical crosslinking via electrostatic interaction with cations, leading to the formation of gel structures [27]. The cation-mediated gelation of FucoPol has been recently reported by Fialho et al. [26], who found that cations such as Fe^3+^ and Cu^2+^ promote the formation of FucoPol hydrogel beads. These structures were prepared by dropping a FucoPol solution (1 wt.%) into an FeCl_3_ solution (1.5 g/L Fe^3+^, 30 mL) through a needle (0.6 mm diameter). The resulting hydrogel beads revealed high stability in deionized water, NaCl 0.9% solution and DMEM at 25 and 37 °C. After immersion for 24 h, no Fe^3+^ was detected in any of the media, which agrees with its revealed non-cytotoxicity properties.

**Figure 1:**
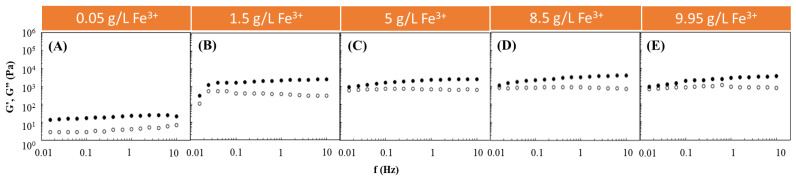
Effect of Fe^3+^ concentration ((**A**) 0.05 g/L, (**B**) 1.5 g/L, (**C**) 5 g/L, (**D**) 8.5 g/L and (**E**) 9.95 g/L) on the storage (G′, solid symbols) and loss (G″, open symbols) moduli of FucoPol HMs prepared using 1wt.% of FucoPol.

Following such studies, the Fe^3+^-mediated gelation of FucoPol was investigated to assess the impact of using different FucoPol and Fe^3+^ concentrations for HM preparation. The effect of Fe^3+^ concentration on the rheological properties of FucoPol HMs was evaluated by combining the FucoPol concentration (1 wt.%) used for preparing hydrogel beads by Fialho et al. [26] with Fe^3+^ concentrations ranging from 0.05 to 9.95 g/L (Figure 1). The selected range was demonstrated to be within the adequate values to promote FucoPol gelation. As shown in Figure 1, for all the tested conditions, the G′ displayed higher values than the G″ over the entire frequency range, thus evidencing the structures’ solid-like behavior and their predominant elastic character [28,29]. Furthermore, both moduli presented a low frequency-independent behavior, which indicates the formation of stable structures [30]. Analogous behavior has been reported for other cation-mediated hydrogels based on polysaccharides such as Ca^2+^-gellan [31], Fe^3+^-succinoglycan [18] and Fe^3+^-xanthan gum [15].

It can be observed that, for an angular frequency of 10 Hz, the lowest G′ and G″ values (0.020 and 0.007 kPa, respectively) were achieved for the lowest Fe^3+^ concentration (0.05 g/L) used (Figure 1A). In this experiment, as the frequency increased, a slight increase in the G″ value was observed that approached the G′ value, indicating the hydrogel’s lower resistance to deformation [32]. However, a significant increase in both moduli was noticed when the Fe^3+^ concentration was raised to 1.5 g/L (Figure 1B). Interestingly, above this concentration, no significant impact on the rheological properties of the structures could be perceived because the G′ and G″ values remained within a range of 0.95–4.0 and 0.60–0.85 kPa (Figure 1C–E), respectively, which are similar to the ones obtained using 1.5 g/L of Fe^3+^ (1.25–2.51 and 0.31–0.55 kPa, respectively) (Figure 1B). The results suggest that a Fe^3+^ concentration of 1.5 g/L is likely enough for good crosslinking of the polymer’s functional groups. Zhou et al. [33] also described an improvement in the rheological properties of poly(ethylene glycol)-based hydrogels modified with dopamine by increasing the Fe^3+^ content in the dopamine:Fe^3+^ ratio from 1:07 to 1:1.

#### 2.1.2. Effect of FucoPol Concentration

**Figure 2:**
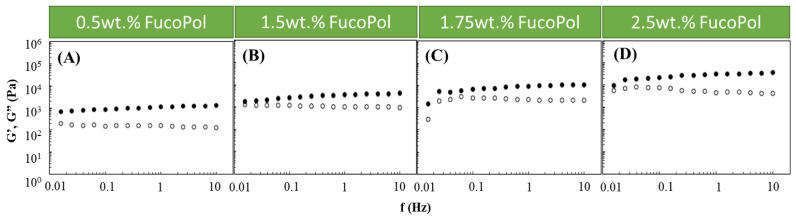
Effect of FucoPol concentration ((**A**) 0.5 wt.%, (**B**) 1.5 wt.%, (**C**) 1.75 wt.% and (**D**) 2.5 wt.%) on the storage (G′, solid symbols) and loss (G″, open symbols) moduli of FucoPol HMs prepared using 1.5 g/L of Fe^3+^.

Based on the obtained results, an Fe^3+^ concentration of 1.5 g/L was chosen to study the effect of the FucoPol concentration on the HM’s rheological properties. Therefore, Fucopol concentrations in the range of 0.5 to 2.5 wt.% were tested (Figure 2). Higher FucoPol concentrations were not possible to test because of the polymer’s high intrinsic viscosity that led to extremely viscous aqueous solutions. As shown in Figure 2, all HMs exhibited a solid-like behavior similar to the ones obtained in the Fe^3+^ effect study (Figure 1). It can be observed that both moduli gradually increased as the polymer concentration rose. Increasing the FucoPol concentration from 0.5 to 2.5 wt.% resulted in an approximately 30-fold increase in the G′ and G″ values (Figure 2A,D). Moreover, using 1.5 and 1.75 wt.% of FucoPol led to an increase in the G′ (1.76–3.99 and 1.50–10.96 kPa, respectively) and G″ (0.96–1.29 and 0.30–2.22 kPa, respectively) values’ ranges (Figure 2B,C). The results demonstrate that the maximum G′ and G″ values (35.3 and 4.2 kPa, respectively) were obtained using the highest FucoPol concentrations (2.50 wt.%), for an angular frequency of 10 Hz (Figure 2D). These values are lower than those reported for Fe^3+^-succinoglycan hydrogel (~70 kPa) [28] and Fe^3+^-xanthan gum (~60/70 kPa) [15] and are significantly higher than those described for Fe^3+^-collagen hydrogels (~0.15 kPa) [34] for the same frequency value. This concentration dependence was also reported for Fe^3+^-xylan hydrogels [35], where a considerable increase in the G′ value (~2.5 to ~10 kPa) was noticed using carboxymethyl xylan amounts of 0.02 and 0.08 g, respectively.

In general, the obtained results demonstrate that both polymer and Fe^3+^ concentrations impact the hydrogel structure, and consequently, FucoPol HM strength can be tuned according to the desired application. Indeed, Fe^3+^ polysaccharide hydrogels comprising varied strengths and elastic properties have been reported as potential structures to be used in applications that include, for example, drug delivery and tissue engineering. For example, Fe^3+^-succinoglycan hydrogels were used as a suitable platform for controlled drug delivery based on gel–sol conversion [18]. Moreover, a polyanionic hydrogel rich in carboxylates and sulphonates crosslinked with Fe^3+^ comprising G′ values between 10^2^ and 10^4^ Pa has been demonstrated to be a promising material in cartilage tissue engineering [36].

Based on the previous experiments, three different FucoPol concentrations (1.0, 1.75 and 2.5 wt.%) combined with 1.5 g/L of Fe^3+^ were selected to prepare FucoPol HMs with distinct rheological properties, which were named HM1, HM2 and HM3, respectively, and were characterized for their physical, chemical and biological properties, as described below.

### 2.2. Morphological Characterization

**Figure 3:**
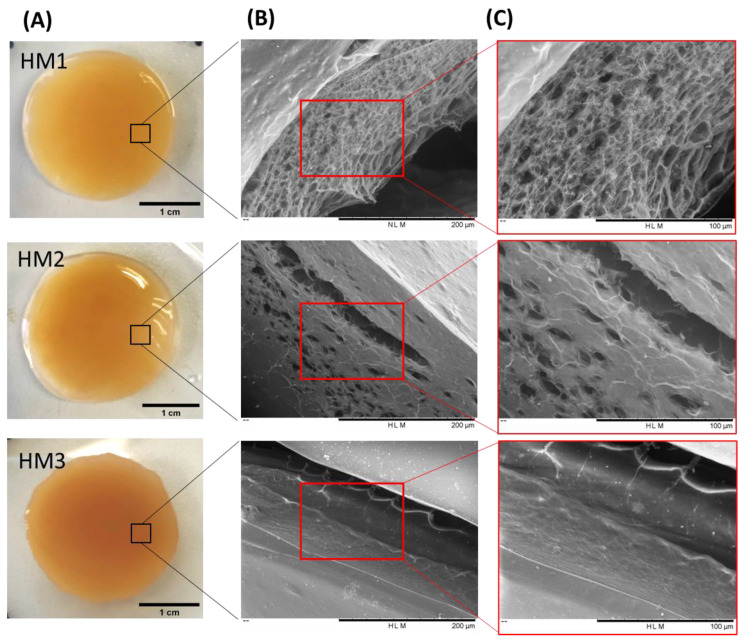
FucoPol HMs: (**A**) macroscopic aspect and corresponding SEM images under (**B**) 500× and (**C**) 1000× magnifications.

As shown in Figure 3A, the three FucoPol HMs exhibited a similar macroscopic appearance, being translucid and displaying an orange coloration, given by the presence of Fe^3+^ cations. However, color intensification and lower translucency were observed for the HM3 membranes, which might be related to their higher polymer and Fe^3+^ contents.

To investigate the surfaces and inner morphology of the FucoPol HMs, SEM observations (Figure 3B,C) were performed. The analysis revealed that all membranes presented a smooth surface, although some structural differences were noticed. The HM1 membranes showed a rather homogeneous three-dimensional network made of polymeric chains. Upon magnification (Figure 3, top panel), it was possible to observe an irregular porous microstructure comprising pores of different sizes. A similar morphology was observed for Fe^3+^-based FucoPol beads prepared with the same polymer concentration [26]. On the other hand, the HM2 membranes displayed a more heterogeneous and much less porous structure, containing several compact regions (Figure 3, center panel). Additionally, for the HM3 membranes, an extremely consistent and dense microstructure could be observed (Figure 3, bottom panel). These results suggest that polymer and Fe^3+^ content significantly impacted the HMs’ microstructure. In fact, an increase in polymer and Fe^3+^ contents in the structures led to a gradual decrease in the porous regions, and the structures became more compact and tighter, likely promoted by higher crosslinking between the polymeric chains and the Fe^3+^ cations. A similar effect was reported for konjac glucomannan hydrogels, which showed a more stable structure and a compact morphology as the Fe^3+^ proportion increased [16].

The porosity of the HMs has an important role in several physicochemical properties of the structures, mainly those related to swelling behavior and drug loading ability [37,38]. As predicted by the SEM observations, the hydrogels’ porosity decreased as the polymer content increased. Therefore, the highest porosity value was observed for the HM1 membranes (59.3 ± 8.3%), and the HM2 and HM3 membranes presented porosities of 22.1 ± 3.1 and 11.8 ± 0.6%, respectively (Table 1). A similar trend was reported for CGC-based [39], chitosan-PVA [38] and alginate-based [40] hydrogels, with the porosity of the hydrogels decreasing for higher polymer and/or crosslinker concentrations.

### 2.3. Chemical Characterization of FucoPol HMs

#### 2.3.1. Composition

HM1, HM2 and HM3 presented polymer contents of 1.75 ± 0.001, 2.28 ± 0.163 and 2.83 ± 0.127 wt.% (Table 1), respectively, which demonstrates that increasing the initial polymer concentration led to the formation of membranes with higher polymer content. Moreover, the same trend was observed for the Fe^3+^ content of the membranes, with HM3 presenting the highest Fe^3+^ content (0.16 ± 0.010 wt.%), and lower values were obtained for the HM1 and HM2 membranes (0.06 ± 0.0002 and 0.10 ± 0.024 wt.%, respectively) (Table 1). These results can be explained by the higher number of anionic groups present in the HM3 hydrogels available to interact with Fe^3+^ cations, increasing their content in the structure.

As listed in Table 1, all FucoPol HMs presented a characteristic high water content [41,42] above 97 wt.%. These results are in accordance with previous work, in which FucoPol hydrogel beads prepared from an initial FucoPol concentration of 1.0 wt.% and gelled with 1.15 g/L Fe^3+^ displayed around 0.15 wt.% of Fe^3+^ and a water content of 98.60 wt.% [26]. The higher Fe^3+^ content obtained in the FucoPol Fe beads might be explained by the high content of FucoPol used in the pyruvyl (13–14 wt.%) and succinyl (3–5 wt.%) groups compared to those presented in this polymer (3.7 and 0.6 wt.%, respectively).

#### 2.3.2. Fourier Transform Infrared (FT-IR) Spectroscopy

**Figure 4:**
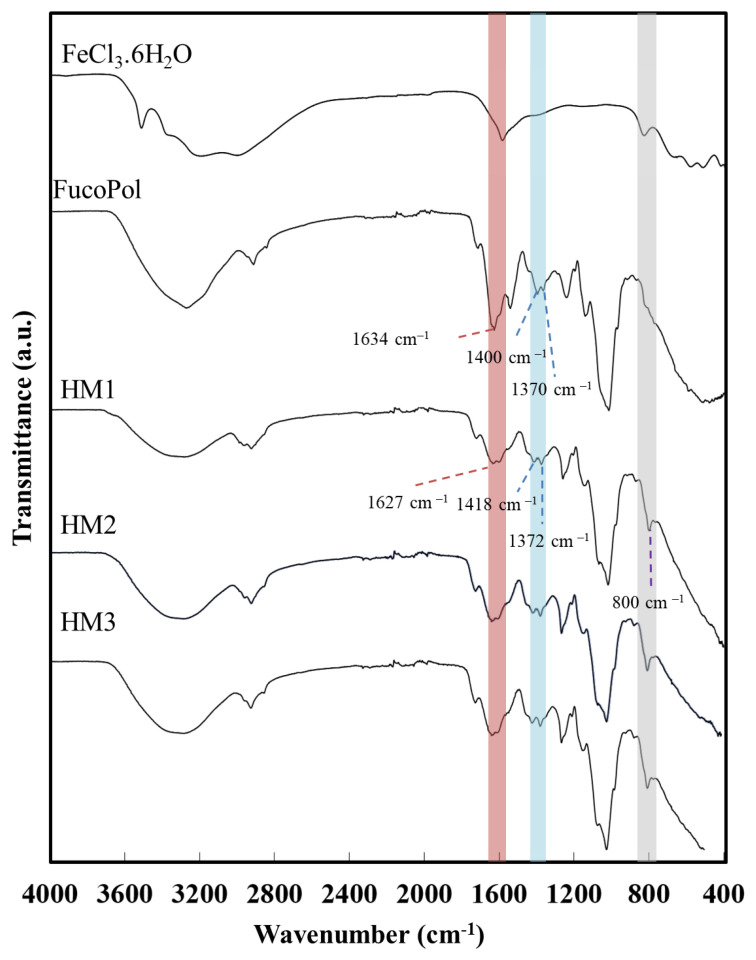
Fourier transform infrared spectroscopy (FTIR) spectra of FeCl_3_·6H_2_O, FucoPol, HM1, HM2 and HM3.

The interaction between the functional groups of FucoPol and the Fe^3+^ cations was assessed via FT-IR analysis. As shown in Figure 4, despite the similarity between the FucoPol and the HMs’ FTIR spectra, the interaction with Fe^3+^ led to a shift in various absorption peaks, mainly those corresponding to the hydroxyl and carboxylate groups.

The intense broadband at 3277 cm^−1^, characteristic of the O–H stretching of hydroxyl groups, and the weak vibration of the C–H stretching peak of CH_2_ groups at 2924 cm^−1^ [43] were shifted to 3300 cm^−1^ and 2927 cm^−1^, respectively. Moreover, the absorption peaks at 1722 cm^−1^ and 1020 cm^−1^, corresponding to the acyl substituents of FucoPol, namely the C=O stretching of carbonyls and the C-O and C-C vibrations of the glycosidic bonds and pyranoid ring [43], respectively, were also shifted to 1720 cm^−1^ and 1016 cm^−1^. The band of C–O–C vibrations of the acyls appearing at 1246 cm^−1^ in the FucoPol spectrum was also shifted to 1259 cm^−1^. The peaks at 1634 cm^−1^ and 1370–1400 cm^−1^, attributed to the asymmetric and symmetric stretching of carboxylates from the glucuronic acid residue [43] were shifted to 1627 cm^−1^ and 1372–1418 cm^−1^, respectively. The wavenumber separation between asymmetric and symmetric stretching vibrations could give information about the binding state of metal cations with –COO^−^ groups [44]. For the HMs’ spectra, wavenumber separations of 255 and 209 cm^−1^ were obtained, which suggest the presence of unidentate binding between Fe^3+^ and –COO^−^ in the hydrogel’s structure [45,46]. Additionally, compared to the FucoPol spectrum, a new peak at around 800 cm^−1^ was noticed in the HMs’ spectra, which might be indicative of the bending of the Fe-O bond [47] from the interaction between the –COO^−^ groups of FucoPol and Fe^3+^ cations.

#### 2.3.3. XRD Analysis

**Figure 5:**
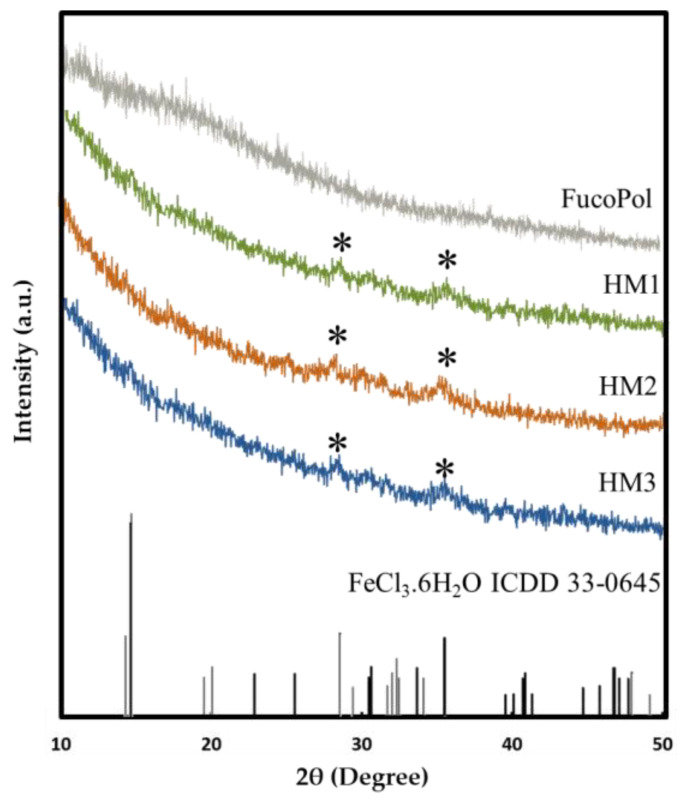
X-ray diffraction patterns of FucoPol and HM1, HM2 and HM3 HMs.

The presence of Fe^3+^ in the structure of FucoPol HMs was also assessed via XRD. As shown in Figure 5, FucoPol presents a diffractogram typical of an amorphous polysaccharide with no characteristic peaks, which is consistent with diffractograms previously reported for FucoPol and other polysaccharides [48]. On the other hand, the diffraction patterns of the HMs exhibited small peaks at around 28.2° and 35.5° (marked with * in Figure 5), which correspond to the (−311) and (221) planes of iron chloride hydrate (ICDD card 033-0645), whose space group is C2/m. This observation confirms the presence of Fe^3+^ cations within the structures [49]. The low intensity of the peaks in the FucoPol HMs’ patterns can be ascribed to the low amount of Fe^3+^ in the structures (Table 1) [49].

### 2.4. Thermogravimetric Analysis

**Figure 6:**
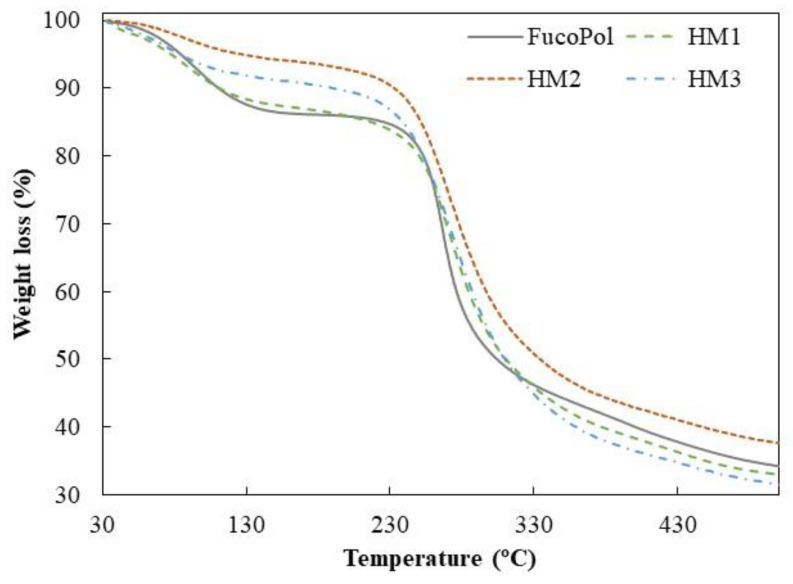
Thermogravimetric curves of FucoPol and HM1, HM2 and HM3 HMs.

Similar TGA curves were observed for all samples, comprising three main thermal degradation steps (Figure 6). In the first degradation step, an increase in temperature from 35–39 °C to 158–170 °C resulted in a weight loss between 6 and 13%, which can be related to the loss of adsorbed and structural water [50]. As shown in Figure 6, up to temperatures of around 230 °C, the HM1 membranes displayed a degradation profile very similar to FucoPol, with similar weight losses (12.7 and 13.4%, respectively) (Table 2). The HM2 and HM3 samples, on the other hand, presented mass losses of 6.0 and 8.7%, respectively, and narrower temperature ranges indicative of a weak water binding capacity [25]. In fact, the higher temperature ranges and the higher weight loss observed for FucoPol and HM1 suggest that they strongly bind to water molecules due to the presence of more functional groups available to establish hydrogen bonds with water. In the HM2 and HM3 structures, those groups are involved in crosslinking with Fe^3+^ cations, which likely decreases the capacity for water binding. An analogous trend was reported by Concórdio-Reis et al. [25], who described a higher weight loss for FucoPol when compared to the biocomposite composed of FucoPol and silver nanoparticles. You et al. [51] also demonstrated that hydrogels of sodium alginate, gelatin and Ca^2+^ improve their thermal stability via the incorporation of copper/tannic acid nanosheets.

The second degradation step, attributed to the polysaccharide decomposition, begins at temperatures of around 164–194 °C, and a 5% mass loss was observed for a temperature range of 234.8–250.7 °C, with a maximum degradation rate from 266 to 272 °C (Table 2). For the FucoPol sample, the second and more significant weight loss (around 39%) occurred between 194 and 324 °C. The thermal degradation profile obtained was similar to that reported for FucoPol as well as for other structures based on FucoPol, such as silver nanocomposites [25] and Fe beads [26]. For the FucoPol HM samples, despite the degradation step starting at lower temperatures (164–186 °C) and higher weight losses being achieved (45.9–52.9%), the mass loss occurred more gradually when compared to the sharp profile obtained for FucoPol (Figure 6). Moreover, those structures presented a Tdeg of 271–272 °C, which is slightly higher than the value displayed by FucoPol (266 °C). These results suggest that the HMs had higher thermal stability, likely given by the crosslinking and coordinate interactions between Fe^3+^ and FucoPol [38]. Enhanced thermal stability was also reported for Fe^3+^-konjac glucomannan hydrogels, which presented a higher Tdeg (275 °C) than those of the other konjac glucomannan-based samples (235–265 °C) [16].

As shown in Figure 6, the gradual mass decrease observed after the second degradation step occurred due to the main-chain scission of the polysaccharide [25] and was identified as the third degradation step. Finally, all samples presented high char yields (31–38%), with the HM2 membranes exhibiting the highest value. Yang et al. [34] attributed this increase to a stronger crosslinking effect. In general, these results show that, although some differences were observed, the thermal properties of FucoPol were not significantly affected by the presence of Fe^3+^.

### 2.5. Mechanical Properties

**Figure 7:**
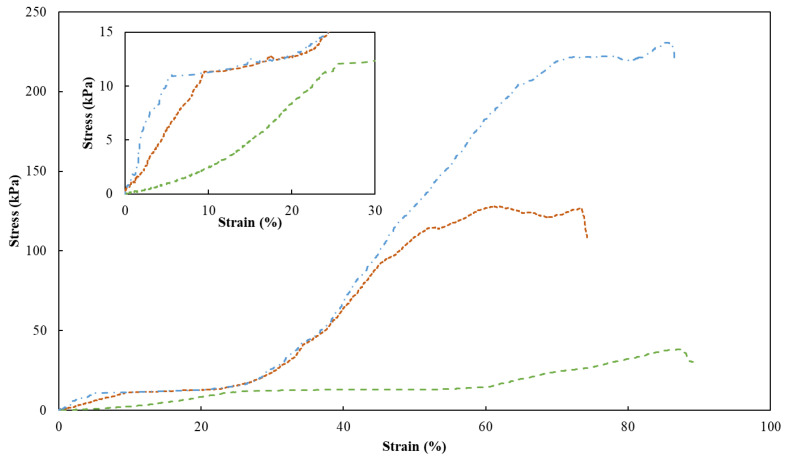
Compression stress–strain curves of HM1 (dashed green line), HM2 (square dotted orange line) and HM3 (dash-dotted blue line) HMs. Insert graph highlights the initial linear deformation of the samples.

To assess the mechanical properties of the FucoPol HMs, a single compression (90% of the initial height) was applied to evaluate the membranes’ hardness, compressive modulus and toughness (Table 1). Figure 7 shows the compressive stress–strain curves of HM1, HM2 and HM3, which demonstrated a linear elastic deformation under small strains (insert graph in Figure 7), followed by a plateau attributed to the deformation of the porous structure via yielding or bending. After that, the denser structure due to the loss of pores resulted in significant strain hardening [52]. It can be noticed that the mechanical characteristics of HMs were significantly affected by their polymer and Fe^3+^ contents. Despite the similarities between the stress–strain profiles, HM1 and HM3 presented similar higher rupture strain values (88% and 86%, respectively), compared to that of the HM2 membrane (around 73%) for the same compressive stress value (90%) (Figure 7).

As presented in Table 1, the highest hardness (221.9 ± 10.2 kPa), compressive modulus (523.3 ± 4.7 kPa) and toughness values (60.7 ± 2.7 kJ/m^3^) were achieved for the HM3 membranes. This result can be related to the higher polymer and Fe^3+^ contents of those membranes (2.83 ± 0.127 and 0.16 ± 0.010 wt.%, respectively) compared to the HM1 and HM2 membranes (1.75 ± 0.001 and 2.28 ± 0.163 wt.%, and 0.06 ± 0.002 and 0.10 ± 0.024 wt.%, respectively) (Table 1). Similar behavior was demonstrated for polyacrylamide/sodium alginate hydrogels, whose mechanical properties were improved by increasing the polymers’ content [53]. Additionally, the introduction of Fe^3+^ in such structures led to the formation of a double crosslinked network, which significantly enhanced the mechanical features of the hydrogels, and as the Fe^3+^ increased from 4.76 to 13.04%, higher values of compressive strengths (495 and 820 kPa) were obtained. Popov et al. [54] also reported higher hardness and compressive modulus values for pectin hydrogels resulting from an increase in the crosslinking cation concentration. For instance, an increase of 24% in hardness was obtained when the Fe^3+^ concentration was increased from 21 to 42 mM [54]. The improvement in the mechanical properties observed for the FucoPol hydrogels is in line and consistent with the SEM observations, which revealed a stronger, compacter and more stable morphology for the HM3 membranes when compared to the HM1 or HM2 membranes (Figure 3).

### 2.6. Water Retention Capacity

**Figure 8:**
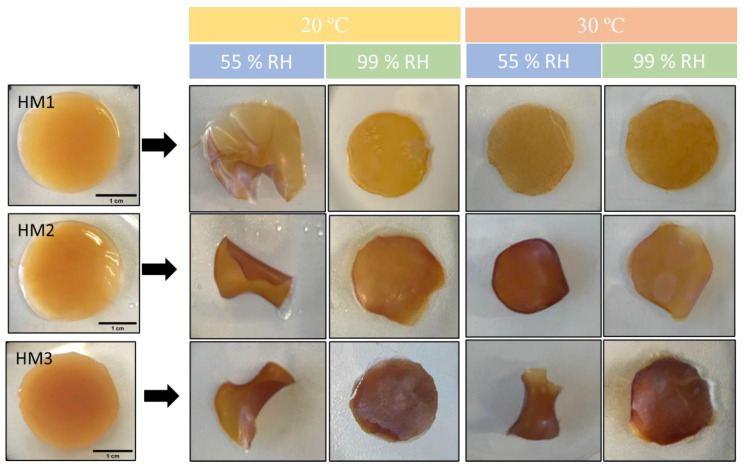
Macroscopic appearance of HM1, HM2 and HM3 HMs samples after being subjected to all the conditions for the study of water retention ability.

The capacity of the FucoPol HMs to retain water inside their structures is determined by the van der Waals forces and hydrogen bonding established between the hydrogels and water molecules [55,56]. This feature plays an important role in several applications. The effect of temperature and relative humidity (RH) in the water retention behavior of the FucoPol HMs was evaluated by incubating samples under RH values of 55 and 99% at temperatures of 20 and 30 °C. The macroscopic appearance of all the membranes is shown in Figure 8. It can be observed that, at the end of each experiment, the HM1 membranes presented a lighter coloration, whereas the HM2 and HM3 membranes intensified their color, and the membranes became brownish. Moreover, under high RH values (99%), all membranes remained flat regardless of the temperature used. On the other hand, under an RH ≈ 55%, likely due to faster water evaporation, wrinkling of the membranes was observed (Figure 8).

**Figure 9:**
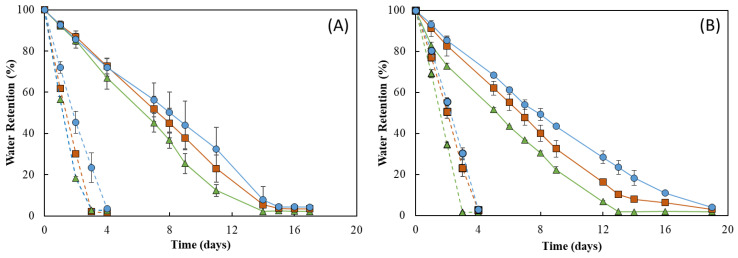
Water retention behavior of HM1 (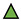), HM2 (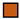) and HM3 (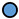) HMs at (**A**) 20 °C and (**B**) 30 °C under 55% (dashed lines) and 99% (full lines) relative humidity.

As shown in Figure 9, the RH strongly influenced the water retention ability of the membranes. In fact, under RH ≈ 55%, all membranes lost water sharply, and after 4 days of incubation, they were dry (water retention ~ 2%) (Figure 9, dashed lines). In addition, 19 days were needed to achieve similar values for incubation carried out at RH ≈ 99% (Figure 9, full lines). On the other hand, no significant differences were perceived for the incubation at 20 °C (Figure 9A) or 30 °C, as similar profiles were observed at both temperatures for all membranes (Figure 9B). However, for all membranes, the water loss rate was higher at 30 °C when compared to that obtained at 20 °C for the same RH value (99%). This behavior of a higher water release with an increase in temperature has also been reported for other polysaccharide hydrogels, such as those based on CMC [57] or chitosan [55].

Figure 9 shows that the water retention ability of the FucoPol HMs was influenced by their polymer and Fe^3+^ contents. For all tested conditions, the HM3 membranes revealed the highest capacity to retain water in their structure, whereas the lowest water retention ability was demonstrated by the HM1 membranes. This difference can be mostly noticed at 30 °C and 99% RH, where HM3 membranes retained approximately 15% more water than that of the HM1 membranes during the experiment. Under those conditions, after 13 days, the HM1 membranes presented a water retention value of 1.9 ± 0.9%, and a significantly higher value (23.5 ± 3.4%) was obtained for the HM3 membranes (Figure 9B). This variation might be explained by the high degree of the crosslinking present in the HM3 membranes, which likely strengthened the physical structure of the hydrogel’s network, promoting water retention inside the structure [58]. Several authors have reported a similar influence of polymer and crosslinker contents on the water retention ability of polysaccharide hydrogels. For example, Kang et al. [55] demonstrated that increasing the concentration of 2,3-dialdehyde cellulose (DAC) from 2.5 to 10% (*w*/*v*) in DAC/chitosan hydrogels led to an increase in their water retention ability from ~30% to ~45% after 11 h at 25 °C. Araújo et al. [39] reported a higher water retention capacity (87.3 to 90.2%) for CGC-based hydrogels with a polymer content of 3.09 ± 0.22 wt.%, compared to 84.6–86.2% for structures with lower polymer concentrations (2.40 ± 0.15 wt.%) after 30 min at 37 °C. Similarly, polyethyleneimine/pectin hydrogels decreased their water retention capacity when the loaded polydopamine/copper nanoparticles were increased from a ratio of 8:0 to 8:2 [59].

### 2.7. Swelling Behavior and Gel Fraction

**Figure 10:**
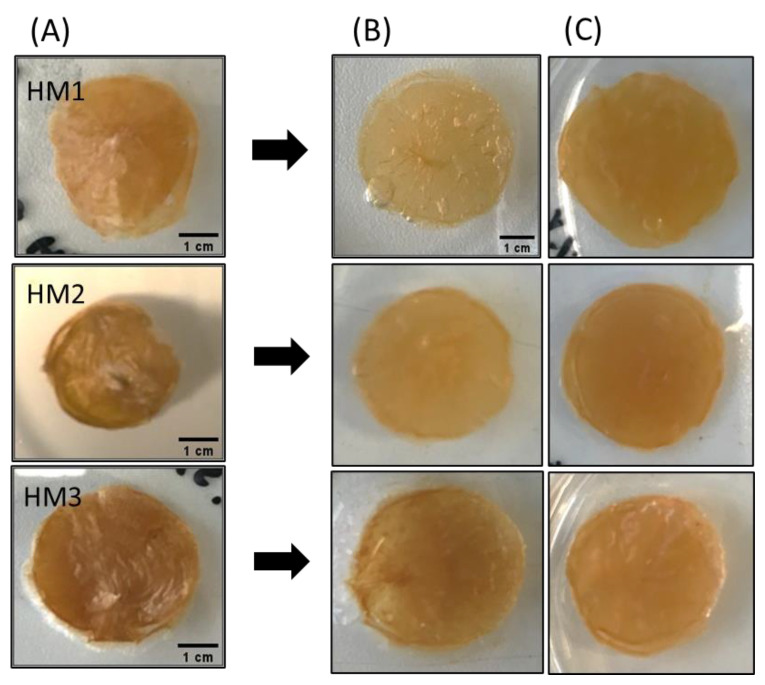
Macroscopic appearance of (**A**) freeze-dried, (**B**) water-swollen and (**C**) NaCl 0.9% swollen HM samples.

The water absorption capability (swelling behavior) of FucoPol HMs was evaluated by immersing previously freeze-dried samples in deionized water or NaCl 0.9% at room temperature for 96 h. Macroscopically, after freeze-drying, the membranes retained their dimensions (Figure 10A), demonstrating that water removal had no significant impact on the polymer’s network. However, the dried membranes presented a lighter brownish coloration, becoming extremely light and brittle. Upon rehydration, besides keeping their dimensions in either medium, all membranes also regained their original orange coloration and translucency. However, the rehydrated membranes were much more fragile (Figure 10B,C).

**Figure 11:**
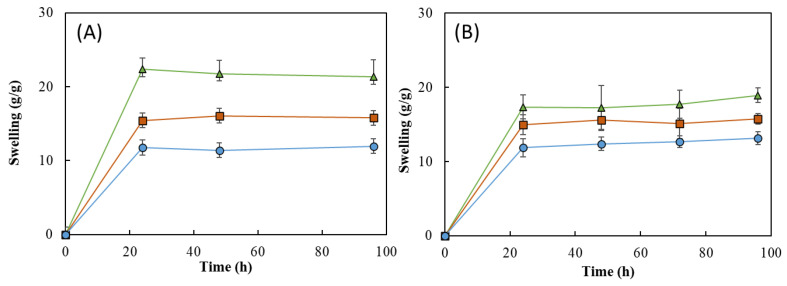
Swelling behavior of HM1 (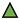), HM2 (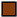), and HM3 (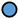) HMs in (**A**) deionized water and (**B**) NaCl 0.9%, at room temperature.

As shown in Figure 11, all the HMs revealed a good water absorption ability, explained by the high hydrophilicity of FucoPol given by the presence of -OH and -COOH groups on its structure [24,53]. It can be noticed that, for all the samples, the swelling equilibrium was achieved in the first 24 h in both media and remained constant thereafter (Figure 11). Upon immersion in the aqueous media for 24 h, the HM1 membranes achieved swelling equilibrium and showed high swelling ratios, as follows: 22.3 ± 1.8 g/g for deionized water and 17.4 ± 1.3 g/g for NaCl. For a similar period, lower swelling ratio values were obtained for the HM2 (15.4 ± 0.3 and 15.0 ± 1.1 g/g) and HM3 (11.8 ± 2.8 and 11.9 ± 1.0 g/g) membranes. These differences can be attributed to an increase in the crosslinking density of those structures compared to the HM1 membranes, which led to a decrease in their ability to sustain water inside the structures [15,60].

Interestingly, the swelling ratio of the HM1 membranes was higher in deionized water (Figure 11A) than in NaCl 0.9% (Figure 11B). After 96 h, the membranes placed in water achieved a swelling ratio of 21.3 ± 1.7 g/g, whereas lower values were obtained in NaCl 0.9% (18.9 ± 0.7 g/g). The decrease in the swelling ability of NaCl 0.9% can be attributed to a lower osmotic pressure difference among the hydrogels and the medium, causing a decrease in the network volume and a shrinkage of the hydrogel. Analogous results were reported for CGC-based [39] and cellulose nanocrystal hydrogels [61].

This behavior was not noticed for the HM2 and HM3 membranes because similar swelling ratios were displayed in water (15.8 ± 0.3 and 11.9 ± 1.6 g/g, respectively) and in NaCl 0.9% (15.8 ± 0.5 and 12.7 ± 0.6 g/g, respectively). These results suggest that the swelling ratio of the HM2 and HM3 membranes is independent of the ionic strength of the solution, which might be explained by the lower size of the structure’s mesh that prevents the shielding of Na^+^ ions from the polymer’s carboxylate groups [62].

**Figure 12:**
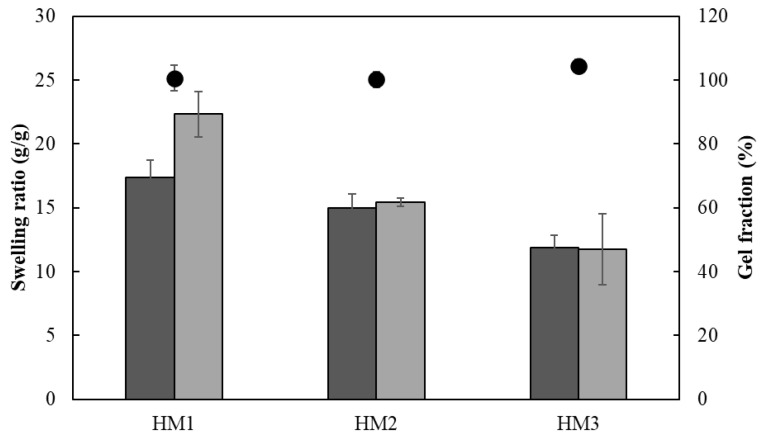
Swelling ratio (columns) after 24 h and gel fraction (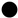) of HM1, HM2 and HM3 HMs in deionized water (light grey) and NaCl 0.9% (dark grey) determined at room temperature.

The ionic crosslinking density of HMs was assessed for the gel fraction study (Figure 12). The gel fraction describes the degree of the crosslinking present in the hydrogel polymer matrix, and its value is inversely proportional to the swelling capacity [63]. In fact, the HM3 membranes presented a slightly higher gel fraction (105.3 ± 2.0%) than that of either HM2 or HM1 (101.4 ± 1.9 and 98.3 ± 1.2%, respectively), which is consistent with the higher polymer and Fe^3+^ contents present in the HM3 membranes (Table 1). Moreover, increasing the crosslinker concentration induced a decrease in the polymer chain relaxation, causing low swelling of the hydrogel [64]. Similar behavior was demonstrated for the Fe^3+^-xanthan gum hydrogels [15], for which the swelling ratio was lowered from 26.92 to 2.31 g/g when the polymer concentration increased from 0.01 to 0.07 g/mL.

### 2.8. Cytotoxicity of FucoPol HMs

**Figure 13:**
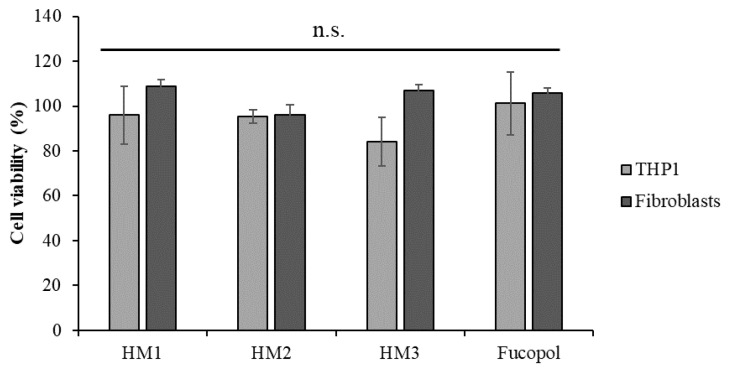
Acute monocytic leukemia cell line, THP1 (light grey bars) and normal dermal fibroblasts (dark grey bars) cell viability (percentage) after 24 h of exposure to HM1, HM2 and HM3 HMs and 500 µg/mL of Fucopol. Bars represent the average ± SD of three independent experiments. n.s.—non-statistically significant.

The cytotoxicity of the HMs was accessed in two human cell lines, an acute monocytic leukemia cell line, THP1, a suspension cell line and normal human dermal fibroblasts, an adherent type of cell line. After 24 h of exposure to each HM, the cells’ viability was analyzed using 3-(4,5-dimethylthiazol-2-yl)-5-(3-carboxymethoxyphenyl)-2-(4-sulfophenyl)-2H tetrazolium and the inner salt (MTS) colorimetric assay, and the percentage of cell viability was calculated after normalization with control cells (untreated). The results show that no statistically significant alterations were detected in the cell viability after exposure to Fucopol HMs compared to the control (Figure 13), demonstrating the non-cytotoxic potential of HMs as previously described by our group for Fucopol [26].

Despite not being statistically significant, there was a slight reduction in the viability of the THP1 cells after exposure to HM3. Indeed, when we compare the physical and mechanical properties of the three HMs (Table 1), we might correlate the slightly higher cytotoxicity of HM3 to its higher Fe^3+^ content, highest hardness and toughness values.

### 2.9. Anti-Inflammatory Activity

**Figure 14:**
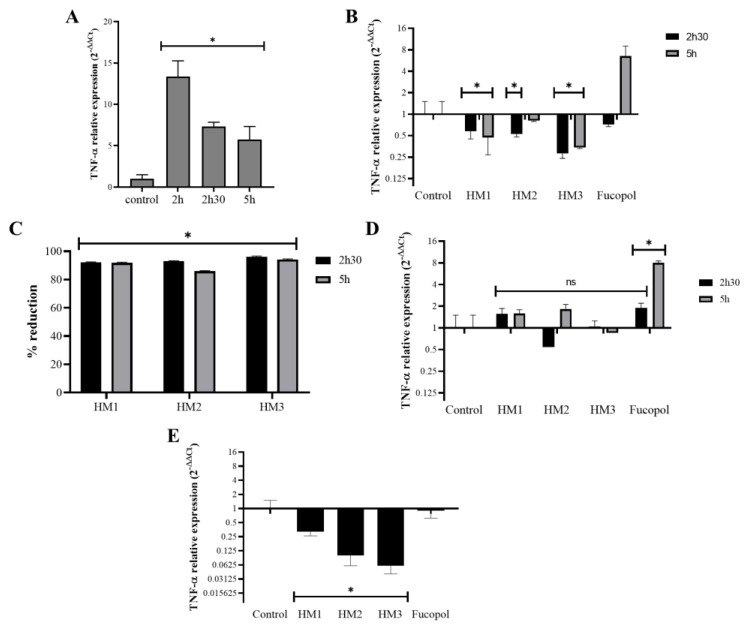
Effect of HMs on TNF-α expression levels in THP1 cells. Inflammation in THP1 cells was stimulated for 2 h via the addition of 7 µg/mL lipopolysaccharides (LPS), and then the HMs or 500 µg/mL of Fucopol were added. In parallel, cells were submitted to the same treatment but without LPS. (**A**) TNF-α expression after 2 h, 2 h 30 and 5 h in LPS-treated samples calculated with 2^−∆∆Ct^ using the *RNA 18S* and corresponding LPS untreated samples as reference. (**B**) TNF-α expression after 2 h 30 and 5 h in LPS-treated samples that were exposed to the HMs and Fucopol for 30 min (2 h 30 samples) and 3 h (5 h samples), calculated with 2^−∆∆Ct^ using the RNA 18S and corresponding LPS control samples as reference. (**C**) Percentage of reduction in TNF-α expression in HM-treated samples relative to the respective control sample (treated with LPS and collected at the same time point). (**D**) TNF-α expression after 2 h 30 and 5 h in LPS untreated samples that were exposed to the HMs and Fucopol for 30 min (2 h 30 samples) and 3 h (5 h samples), calculated with 2^−∆∆Ct^ using the *RNA 18S* and corresponding control samples as reference. (**E**) TNF-α expression after 2 h in samples that were simultaneously submitted to LPS and to the HMs or Fucopol, calculated with 2^−∆∆Ct^ using the *RNA 18S* and corresponding LPS untreated samples as reference. Bars are the average ± SEM of three independent experiments. * *p*-value below 0.05 relative to control; ns—non-statistically significant.

The potential anti-inflammatory activity of the Fucopol HMs was examined in acute monocytic leukemia cells, THP1, after or simultaneously to their exposure to an inflammatory stimulus caused by a bacterial lipopolysaccharide (LPS) by analyzing the expression levels of the proinflammatory cytokine tumor necrosis factor alpha (TNF-α) (Figure 14). As expected, TNF-α expression in samples incubated for 2 h with LPS showed a very high level (12× compared to the control) that reduced after 2 h 30 and 5 h of incubation, but it remained higher than that of the control (≅5.5×) (Figure 14A). Interestingly, the addition of the Fucopol HMs to THP1 cells 2 h after the stimulus with LPS resulted in a decrease in TNF-α expression (Figure 14B) of more than 80% for all membranes when compared to the control samples (incubated with LPS) (Figure 14C). Fucopol was able to induce a reduction in TNF-α expression after 2 h 30 but not after 5 h of exposure (Figure 14B). When samples were not previously stimulated with LPS, no statistically significant alterations were detected in the TNF-α expression of the cells exposed to the membranes, as opposed to a statistically significant increase in the TNF-α expression when cells were incubated with Fucopol (Figure 14D). Moreover, the simultaneous exposure of THP1 cells to LPS and Fucopol HMs resulted in the decreased expression of TNF-α when compared to cells only incubated with LPS, with a higher reduction observed when cells were incubated with HM3 (Figure 14D), indicating that the high level of iron content and mechanical characteristics of this membrane could indicate their more suitable application to avoid inflammatory processes. In general, these results show the anti-inflammatory potential of the HMs in cells previously stimulated with an inflammatory agent or during the inflammatory process, which might be a highly positive feature for the treatment of inflammatory diseases or to control inflammatory processes.

## 3. Materials and Methods

### 3.1. Materials

FucoPol was obtained via cultivation of the bacterium *Enterobacter* A47 (DSM 23139) in a 10 L bioreactor (BioStat B-plus, Sartorius, Germany) using glycerol as the sole carbon source, as described by Concórdio-Reis et al. [65]. FucoPol was recovered and purified from the cultivation broth via diafiltration and ultrafiltration, as previously described [25]. FucoPol was composed of fucose (36% mol), glucose (33% mol), galactose (26% mol) and glucuronic acid (5% mol), with a total acyl group content of 7.8 wt.%. The polymer presented a number (Mn) and molecular weight (Mw) of 1.68 × 10^6^ Da and 3.19 × 10^6^ Da, respectively, with a 1.90 polydispersity index. The sample had a protein and inorganic salt content of 14.3 and 1.4%, respectively.

### 3.2. Preparation of FucoPol HMs

**Figure 15:**
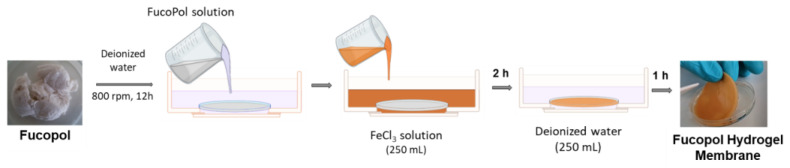
Schematic illustration of Fucopol HM preparation, comprising polymer dissolution in deionized water, immersion in FeCl_3_ solution for polymer gelation and washing step.

FucoPol HMs were fabricated as shown in Figure 15. Freeze-dried FucoPol was dissolved in deionized water (1 wt.%) under magnetic stirring (800 rpm) at room temperature until complete dissolution. The resulting FucoPol solution was cast into a silicone cylindrical mold (50 mm diameter, 3 mm height), and the height was leveled to the mold using a spatula. HMs were prepared via immersion of the silicone mold with the FucoPol solution into an aqueous FeCl_3_ solution (250 mL) at room temperature for 2 h. Afterward, the hydrogel membranes were washed with deionized water (250 mL) for the removal of the unreacted crosslinker. After 1 h, FucoPol HMs were removed from the mold, and their rheological properties were evaluated via oscillatory shear measurements. Different Fe^3+^ (0.05–9.95 g/L) and FucoPol (0.5–2.5 wt.%) concentrations were tested to assess their impact on the strength of the HMs.

### 3.3. Rheological Properties

The rheological behavior of FucoPol HMs was determined using a modular compact rheometer (MCR92, Anton Paar, Graz, Austria), equipped with a parallel plate geometry (diameter 20 mm) with a 1 mm gap. HM samples with 25 mm of diameter and similar thickness (~2 mm) were equilibrated at 25 °C for 5 min. The viscoelastic properties were assessed by applying frequency sweeps at a constant tension within the linear viscoelastic region for a frequency range from 0.01 to 10 Hz.

### 3.4. FucoPol HMs Characterization

Based on the previous results, three different concentrations of FucoPol (1.0, 1.75 and 2.5 wt.%) were selected to be combined with Fe^3+^ at a concentration of 1.5 g/L. The obtained membranes were labeled as HM1, HM2 and HM3, respectively. The prepared HMs were cut with a stainless steel cylindrical mold (25 mm diameter) to promote better handling. Additionally, to remove all uncrosslinked Fe^3+^, smaller HM samples were washed with deionized water (250 mL) under continuous stirring (150 rpm) until constant conductivity (~1 µS/cm) was achieved.

#### 3.4.1. Chemical Characterization

The water content (%) of the FucoPol HMs was determined gravimetrically by freeze-drying the samples, using following the equation:

Water content = (W_Wet_ − W_dry_)/W_wet_ × 100, (1)

where W_dry_ (g) represents the dry mass of a pre-weighed amount of the HM (W_wet_, g).

For the quantification of the HMs’ iron content, freeze-dried FucoPol HM samples (~5 mg) were hydrolyzed with nitric acid (5 mL HNO_3_ 5% *v*/*v*) at 120 °C for 2 h. Hydrolyzed samples were filtered, and their iron content was determined via Inductively Coupled Plasma-Atomic Emission Spectrometry (ICP-AES) (Horiba Jobin-Yvon, France, Ultima model) equipped with a 40.68 MHz RF generator, Czerny–Turner monochromator with 1.00 m (sequential), autosampler AS500 and Concomitant Metals Analyzer (CMA). Nitric acid solution (HNO_3_ 5% *v*/*v*) was subjected to the same hydrolysis procedure and used as blank.

#### 3.4.2. Morphology

The structure and morphology of the FucoPol HMs were investigated via scanning electron microscopy (SEM) using Tabletop Microscope TM3030 (Hitachi in High Technologies, Tokyo, Japan) equipped with a refrigerated sample holder. Wet HM samples were placed in the sample holder, and small cuts were performed on its surface using a scalpel to promote better observation of the inner microstructures. The observations were performed at −4 °C using magnifications of 500× and 1000×.

#### 3.4.3. Porosity

The porosity of the FucoPol HMs was evaluated via the solvent replacement method. Briefly, pre-weighed freeze-dried HM samples (W_0_, g) with 13 mm diameters were immersed in absolute ethanol for 30 min. After that, the excess ethanol was removed, and the samples were weighed (W_30_, g). The porosity (%) was determined using the following equation:

Porosity = (W_30_ − W_0_)/(ρV_T_), (2)

where ρ is the density of ethanol (0.790 g/cm^3^) and V_T_ (cm^3^) is the total volume of the HM sample.

#### 3.4.4. Fourier Transform Infrared (FT-IR) Spectroscopy

FucoPol and FucoPol HMs were characterized via Fourier-transform infrared spectroscopy (FT-IR) with a spectrum two spectrometer (PerkinElmer, Waltham, MA, USA) equipped with the attenuated total reflectance (ATR) accessory. The spectra were recovered based on five scans between a resolution of 4000 and 400 cm^−1^ at room temperature.

#### 3.4.5. X-ray Diffraction (XRD) Analysis

The crystalline structure of FucoPol and the FucoPol HMs was studied/analyzed using a diffractometer (X’Pert Pro, PANalytical, Almelo, The Netherlands) with a monochromatic Cu Ka radiation source with a wavelength of 1.5406 Å. Measurements were carried out from 10 to 50° (2θ) with a scanning step size of 0.016° in the continuous scanning mode.

#### 3.4.6. Thermogravimetric Analysis (TGA)

The TGA of FucoPol and FucoPol HMs was performed using thermogravimetric analyzer Setaram Labsys EVO (Setaram, Sophia Antipolis, France) in a temperature range from room temperature to 500 °C with a heating rate of 10 °C/min under an argon atmosphere. The 5% weight loss (T5%, °C) was attributed to the temperature at which the initial 5% of the mass was lost, and the thermal degradation temperature (Tdeg, °C) was assigned to the temperature value obtained for the maximum decreasing peak of the sample mass.

### 3.5. Compressive Mechanical Analysis

The compressive mechanical properties of the FucoPol HMs were assessed with a texture analyzer TMS-Pro (Food Technology Corporation, England) equipped with a 250 N load cell. HM samples with a thickness of 2–3 mm were cut into a cylindrical shape (25 mm diameter) and were compressed up to 50% strain of the original height at a speed rate of 60 mm/s using a plunger with a 37 mm diameter. The maximum tension of the compression corresponds to the hardness (kPa) and the toughness (kJ/m^3^), calculated by measuring the area underneath the stress–strain curve of each sample. The compressive modulus (kPa) was obtained as the slope of the initial linear region. All the experiments were performed at room temperature.

### 3.6. Water Retention Capacity

To determine the water retention capacity of FucoPol HMs, cylindrical samples (25 mm diameter, 2 mm thickness) were placed in a desiccator with two different saturated salt solutions, magnesium nitrate (Mg(NO_3_)_2_) and potassium sulfate (K_2_SO_4_), to provide relative humidity (RH) values of 55% and 99%, respectively. Samples were taken out from the desiccator at specific intervals and were weighed (W_t_). The influence of temperature was also evaluated by performing the experiments at 20 °C and 30 °C. Water retention (%) was determined with the following equation:

Water retention = W_t_/W_wet_ × 100, (3)

where W_wet_ represents the initial weight of the HMs.

### 3.7. Swelling Behavior

The swelling properties of the FucoPol HMs were assessed gravimetrically by immersing pre-weighed (W_dry_, g) freeze-dried samples in deionized water and NaCl 0.9% (*v*/*v*) at room temperature for 96 h. At different time intervals, samples were carefully taken out from the solutions, the excess water was removed using filter paper, and samples were weighed (W_t_, g). The swelling ratio (g/g) was calculated as

Swelling ratio = (W_t_ − W_dry_)/W_dry_, (4)

### 3.8. Gel Fraction

For the determination of the gel fraction, which represents the crosslinking density in the hydrogel structure, freeze-dried samples of the HMs were immersed in deionized water at room temperature for 72 h. After that period, rehydrated HM samples were freeze-dried again, and the gel fraction (%) was determined using the following equation:

Gel fraction = W_f_/W_dry_ × 100, (5)

where W_f_ and W_dry_ represent the dry weight (g) of HM samples after and before immersion in deionized water, respectively.

### 3.9. Biological Assays

#### 3.9.1. Cell Culture and Culture Media

Biological assays were performed with two cell lines acquired from American Type Culture Collection (ATCC, Manassas, VA, USA): human primary dermal fibroblasts (PCS-201-010) and human leukemia monocytic cell line THP1 (TIB-202). Fibroblasts cells were cultured in Dulbecco’s modified Eagle medium (DMEM, Thermo Fisher Scientific, Waltham, MA, USA) supplemented with 10% (*v*/*v*) fetal bovine serum (FBS, Thermo Fisher Scientific) and a mixture of 100 U/mL of penicillin and 100 µg/mL of streptomycin (Thermo Fisher Scientific). THP1 cells were cultured in Roswell Park Memorial Institute medium (RPMI 1640, Thermo Fisher Scientific) supplemented with 10% (*v*/*v*) FBS, 1x MEM Non-Essential Amino Acids Solution (Thermo Fisher Scientific) and a mixture of 100 U/mL of penicillin and 100 µg/mL of streptomycin (Thermo Fisher Scientific).

#### 3.9.2. Cytotoxicity Tests

For the cell viability experiments, fibroblasts cells were seeded in a 24-well plate at a concentration of 1 × 10^5^ cells per well and were incubated at 37 °C, 5% (*v*/*v*) CO_2_ and 99% (*v*/*v*) relative humidity. After 24 h for cell adherence, samples of FucoPol HMs (5 mm diameter) were added to a well with cells and to a well without cells, which were used as a control. Upon 24 h of exposure, the samples were removed, and the medium was replaced with a fresh medium (500 µL) supplemented with MTS, incubated (37 °C, 5% (*v*/*v*) CO_2_) for 45 min. THP1 cells were cultured in a 24-well plate at a density of 1 × 10^5^ cells per well, and samples of HMs (5 mm diameter) or 500 µg/mL Fucopol were added at the same time as cell seeding. Upon 24 h of exposure, the Cell Titer 96^®^ Aqueous One solution cell proliferation assay (Promega, Madison, WI, USA) (200 µL) was added to the medium and incubated (37 °C, 5% (*v*/*v*) CO_2_) for 60 min. Following incubation, the absorbance was measured at 490 nm according to the manufacturer’s instructions. The cell viability of primary fibroblasts and THP1 exposed to FucoPol HMs was expressed in a percentage and was calculated using the following equation:

(6) $$\%\;viability=\frac{Abs_{490}\;fibroblasts\;with\;FucoPol\;HM-Abs_{490}\;medium\;with\;FucoPol\;HM}{Abs_{490}\;fibroblasts-Abs_{490}\;medium}\times 100$$

#### 3.9.3. Anti-Inflammatory Activity

The analysis of the anti-inflammatory activity of the Fucopol HMs in THP1 cells proceeded as previously described with a few modifications [66]. Briefly, 1 × 10^6^ THP1 cells were seeded in 6-well plates, incubated for 2 h with 7 µg/mL Lipopolysaccharide (LPS, Sigma Aldrich, St. Louis, MO, USA) and were then incubated for 30 min or 3 h with the Fucopol HMs (13 mm diameter) or with 500 µg/mL of Fucopol. Identical samples without the addition of LPS were prepared (-LPS) for negative control purposes. In another approach, cells were simultaneously exposed for 2 h to both LPS and the Fucopol HMs or to 500 µg/mL of Fucopol. After the incubation time, cells were pelleted via centrifugation (500× *g*, 5 min) and solubilized in 300 µL of NZYol (NZYtech, Lisboa, Portugal), and RNA was extracted according to the manufacturer’s instructions and reverse-transcribed with the NZY M-MulV First strand cDNA synthesis kit (NZYtech). The relative expression of the tumor necrosis factor α gene (TNF-α) and of the housekeeping gene *RNA18S* were determined using the Ct method (2^−∆∆Ct^) [67] after real-time quantitative amplification using the NZYSupreme qPCR Green Master Mix (NZYtech) in a Corbett Rotor-Gene thermal cycler (Qiagen, Hilden, Germany).

### 3.10. Statistical Analysis

The experimental data from all the studies were analyzed, and the results are expressed as mean ± standard deviation (SD). Error bars represent the standard deviation (n ≥ 3). Differences between results were considered statistically significant when the *p*-value was <0.05.

## 4. Conclusions

This study demonstrates for the first time the preparation of hydrogel membranes based on the polysaccharide FucoPol, using Fe^3+^ as a crosslinking agent. From oscillatory shear measurements, it was shown that the FucoPol concentration had a significant impact on the HMs’ strength. Regarding the effect of the Fe^3+^ concentration, for values above 1.5 g/L, a minimal effect was observed. Three FucoPol concentrations (1.0, 1.75 and 2.5 wt.%) were selected to be combined with Fe^3+^ (1.5 g/L), whose presence was confirmed via structural analysis. Despite the high water content of all the prepared HMs, their morphology and their mechanical, water retention and swelling properties were affected by their composition. Moreover, HMs did not show cytotoxic activity in two different human cell lines (leukemia cells line and normal primary human fibroblasts) but showed anti-inflammatory capability in cells previously stimulated with an inflammatory agent or during the inflammatory process, which might be a highly positive feature for the treatment of inflammatory diseases or to control the development of inflammatory processes. Overall, this study demonstrates the promising potential of FucoPol as a versatile material for HM fabrication, offering tunable properties, biocompatibility and anti-inflammatory activity.

## Figures and Tables

**Table 1:** Physical and chemical characterization and mechanical properties (under 90% strain) of FucoPol HMs prepared from the gelation of FucoPol with Fe^3+^ (1.5 g/L) using polymer concentrations of 1.0 wt.% (HM1), 1.75 wt.% (HM2) and 2.5 wt.% (HM3).

Sample ID	HM1	HM2	HM3
Polymer content (wt.%)	1.75 ± 0.001	2.28 ± 0.163	2.83 ± 0.127
Fe^3+^ content (wt.%)	0.06 ± 0.002	0.10 ± 0.024	0.16 ± 0.010
Water content (wt.%)	98.19 ± 0.001	97.62 ± 0.187	97.02 ± 0.138
**Mechanical properties**			
Hardness (kPa)	32.4 ± 5.8	131.1 ± 6.5	221.9 ± 10.2
Compressive modulus (kPa)	56.3 ± 7.8	353.3 ± 24.9	523.3 ± 4.7
Toughness (kJ/m^3^)	1.4 ± 0.1	34.5 ± 5.5	60.7 ± 2.7

**Table 2:** Thermal degradation steps and degradation temperature (Tdeg) of FucoPol and FucoPol HMs.

	1st Degradation Step		2nd Degradation Step				Char Yield (%)
Sample	Temperature Range (°C)	Weight Loss (%)	Temperature Range (°C)	Weight Loss (%)	T5% (°C)	Tdeg (°C)	
FucoPol	37–166	13.4	194–324	39.2	250.7	266	34
HM1	36–170	12.7	186–370	45.9	244.8	271	33
HM2	39–162	5.9	174–374	49.0	240.0	272	38
HM3	35–158	8.5	164–378	52.9	234.8	272	31

## Data Availability

Data is contained within the article.
